# Remarks on the Qualifications of Apothecaries

**Published:** 1813-12

**Authors:** 


*To the Editor of the Medical and Physical Journal.*

SIR,

THE memoir on Medical Reform, by a disinterested Physician, in your last Journal, certainly contains some very excellent observations, but also many incongruities. He sets out with stating the importance of securing to society a sufficient number of duly-qualified practitioners, who shall be able to accommodate themselves to the various classes and conditions of the community, so as to afford to each the best assistance that the present state of medical science will admit of, and which he deems must be admitted by all to be a matter of grave and deep importance. And though he thus expresses himself very desirous to improve the professions of physic and surgery, and thereby to benefit the public, yet is he decidedly hostile to the measures at present in contemplation for the attainment of those important objects, although they appear admirably calculated for the purposes intended.

He then proceeds to specify the several kinds of medical practitioners who are to be found at the present day dispersed throughout the British islands, and afterwards comments on each of their characters, in which the apothecary comes in for no small share of censure, although he is now and then admitted to be an useful character, and found to be as deservedly trusted as those who move in the higher departments of the profession, even under the auspices of a university degree, as the following observations, indeed, would almost tempt one to believe was true.

Speaking of the degradation and neglect to which physicians are exposed, the author of the memoir asks, "May they not be distinctly traced to the numerous and discordant sources from whence the physicians have been derived; to the want of suitable education being afforded by those seminaries, to whose graduates, notwithstanding, the greatest privileges are by law established; and, finally, to the corrupt practice of other universities, in granting degrees on private testimonials of qualification only, which testimonials are too often obtained by private favor, or gross venality, and granted to individuals whom no university ought, consistently with a due regard to its own reputation, to acknowledge? The excess to which this latter system of doctor-making is carried, or the systematic corruption by which certificates are obtained, is far from being generally imagined; and I speak from certain knowledge of the facts, when I allege that whoever can offer a colorable pretext for making an application to Aberdeen or St. Andrew's, need not be debarred from his degree by any difficulty in finding *physicians ready and willing to grant the necessary certificates*, provided he is prepared to make the pecuniary recompense required. And the *trade* seems to be *both prosperous and lucrative*, for a *recently made* doctor of this class, whose university fees at St. Andrew's amounted to only 24*l.*, had his total expences increased by such contingencies as I am alluding to, to 150*l.* Further, it is only of late that such gross venality and infamous corruption in the profession of physic have come to my knowledge: I have oftentimes, indeed, heard such allegations advanced, but contemned them as groundless calumnies. I have now, however, had but too good proof for asserting, that the granting of certificates to candidates for Aberdeen or St. Andrew's degrees in physic, is become a matter of *regular barter and trade*; and that a sufficiency of money is all that is required to advance any who has ever dabbled in medicine to the disgraced and unenviable rank of M.D."

Surely there is no regular-bred apothecary in the kingdom who who would suffer by a comparison with a physician who has obtained his academic honors in this manner, and not one of the former but what is more justly entitled to public confidence. And though the writer of the memoir does admit, "that among apothecaries may be found individuals possessed both of learning and abilities, and who, by their association, would reflect credit on any College, whether of Surgeons or Physicians;" yet he is by no means desirous that any plans should be adopted for securing to the public a greater number, and a continuance of such characters; but on the contrary he is only anxious for their entire extinction, as though they were so many excrescences to the profession, notwithstanding the above admission, that there are men to be found among them of acknowledged merit, and likewise without any proof that the regular apothecary is generally more deficient than the rest of his medical brethren, or less worthy to be trusted than the physicians above described.

The same writer, however, does not appear to be aware that the regular-bred or legitimate apothecary of this country is a very different character from the many who pretend to this appellation: he is understood to be a man who, having served an apprenticeship to the immediate business of an apothecary, afterwards perfects himself in a knowledge of diseases, by an attendance on lectures, dissections, and hospital practice, although he may afterwards confine himself to the mere business of an apothecary; and this is the education that a respectable practitioner of this kind is always expected to have had. And that no persons, but those so educated, and who shall afterwards undergo examination to give proof of their ability, be allowed to practise as apothecary or surgeon-apothecary, is the object sought by the intended application to parliament; and, without the adoption of some such measures, there can be no security to the public of having a qualified body of practitioners of any kind. For even according to the statement of the disinterested Physician, as may be seen above, there are very frequently imperfections and abuses in the medical educations of physicians, as well as in those of apothecaries; and although the surgeon, who has his diploma from Surgeons' Hall, is the only character in his eyes fitted for the practice of physic as well as surgery, yet, as he admits that no *medical* examination is imposed on the surgeon when he obtains a diploma, I cannot conceive that there is any security given to the public of his acquaintance with the practice of physic; though, notwithstanding this, the same writer asserts, that a surgeon starting under the auspices of the Royal College of Surgeons, geons, must therefore necessarily be better acquainted with pharmacy and the practice of physic than either the physician or apothecary, though it is notorious that some of the most eminent surgeons in town, and even public teachers of surgery there, are oftentimes wholly unacquainted with the practice of physic, and, moreover, it is no uncommon thing with them to speak of it in terms of contempt. The two professions are perfectly distinct, and I know not how a young man that is brought up to surgery *only*, can know physic more, unless intuitively, than one bred to physic can know of surgery; and, notwithstanding the sneers cast at apothecaries for uniting in themselves the practice of surgery and pharmacy, I believe them to be just as well qualified for the purpose, as any other member of the profession is who has only qualified himself for a particular branch.

The mere surgeon, surely, who undertakes the practice of physic and pharmacy without a suitable knowledge of these branches, is just as deserving of contemptuous treatment as the apothecary who without an adequate knowledge undertakes surgery, and there is generally as much danger to the public in a deviation from the particular practice of the one as the other; and the only security that can ever be afforded to the public of having medical men capable of acting with ability in the united capacities of surgeon and apothecary, will be, by the grant of certificates, by some superintending institution, to those who are found qualified for the combined practice.

If the members of the profession eould but be persuaded to lay aside their feuds and jealousies, and would unite in the adoption of some plan for preventing all uneducated and unqualified persons from practising in any branch of the profession, such a measure would give to the latter all the improvement that it is susceptible of, and to men of merit a much wider field of practice than they will ever have without some such regulation. It is in vain to hope for improvement without it, by putting down these or those particular men of the profession, because the other branches of it would become stocked in proportion; for instance, if there were to be no apothecaries, there would then be a greater number of surgeons and physicians; and therefore the latter persons would experience no relief from the removal of the former, although an object so much aimed at in the present day; and I am convinced that the only relief that can ever be given to the profession generally, will be by the prevention of all irregular persons from entering into it.

It is admitted by the writer of the memoir on medical reform, that a degree of doctor of physic from either of the universities universities of Oxford and Cambridge, is not any proof th a the individual on whom it is conferred is therefore fully competent to the practice of physic, and worthy to be trusted with the lives of the community; for he observes, "To neither of these universities does any efficient school of physic belong. They confer medical degrees, however; but rather as being arrived at in the regular course of academic discipline, and attained by a certain observance of acts and terms, than as merited by any full or perfect qualifications in the art of curing diseases; yet these graduates possess privileges such as no other medical men enjoy, and are entitled to demand admission as fellows of the London College of Physicians, without undergoing the scrutiny of an examination, to which all other candidates are subjected."

We know also pretty well, from the same writer, how degrees are obtained from the universities of Aberdeen and St. Andrew's; and perhaps there are very few other universities, (for the accounts of the former ones are apt to make one suspicious,) from whom a doctor's degree is any proof that the person possessing it has been sufficiently educated, and has really studied during a university period all the branches of medicine. And notwithstanding too all that has been said in favor of the person, as a general practitioner, who has a diploma from Surgeons' Hall, yet such a person undergoes no examination in medicine or pharmacy, nor is obliged to give any proof of his acquaintance with either.

From what, therefore, has been stated, I really think the regular apothecary is as justly entitled to confidence as any of his medical brethren, although their names may possibly be of a more imposing kind.

But I should suppose that it must appear pretty evident to all that have duly considered the matter, that the only way to improve the profession, and at the same time to secure to the public a regular supply of competent practitioners, will be, by the imposition of such restraints as will prevent any individual from practising in any one department of it, who has not had a suitable medical education; and who should afterwards, before he be admitted to practise, give further proof of his medical or surgical attainments, or of both, by an examination before competent judges: and I know of no better way for the accomplishment of all this than in the manner proposed by the new bill of the London Committee of Apothecaries and Surgeon-apothecaries, to the support of which I would therefore earnestly recommend every sincere friend to the profession.

The writer of the memoir on medical reform, has, in reality, proposed no reform, or at least nothing that appears to me calculated to remove the evils under which the profession labors. He has, indeed, pointed out how improperly many universities grant degrees in physic, and how frequently unqualified apothecaries act in all departments of physic and surgery; likewise how much better it would be for the surgeon to unite in himself all the branches of physic, surgery, midwifery, and pharmacy, than for any other individual to do so: but not one word has he said about the advantages that would result both to the public and to the profession, from the exclusion of uneducated persons;—and I do really believe that if all such were in future to be prevented from practising either in pharmacy, physic, or surgery, together with the necessity that all persons should undergo examination before they were allowed to practise, that then the professions of physic and surgery would receive all the improvement they are capable of admitting, and the most beneficial consequences would soon result to the public.

The Board of the National Vaccine Establishment of London, in their last report, remark, with great concern, that the mortality from small-pox has this year increased very considerably; and among the causes which have conspired to occasion it, they allude to the practice of inoculation for that disease daily carried on in the metropolis by a number of *unauthorised* practitioners. This is one among the many striking proofs of the mischief done by suffering those persons to practise whom I have been reprobating, and is likewise a further confirmation of my opinion of the necessity of such legislative interference as will keep unqualified persons out of a profession, in which is oftentimes placed the power of conferring or taking away life. The injury occasioned by continuing the infusion of small-pox alone is incalculable, and to use the words of the Board above-mentioned, "is continuing a disease which has for centuries been no less detrimental to the population of states, than prejudicial to the health of mankind."

*Bristol, Oct.* 20, 1813. An APOTHECARY.

